# Effect of high-intensity interval training on aerobic capacity and fatigue among patients with prostate cancer: a meta-analysis

**DOI:** 10.1186/s12957-022-02807-8

**Published:** 2022-10-19

**Authors:** Ming Chang, Junguo Wang, Hairul A. Hashim, Shihao Xie, Adam A. Malik

**Affiliations:** 1 Exercise and Sports Science Programme, School of Health Sciences, Universiti Sains Malaysia, Kubang Kerian, Kelantan 16150 Malaysia; 2 Department of Orthopaedics, Qingdao Hospital of Traditional Chinese Medicine (Hiser hospital), Qingdao, Shandong China

**Keywords:** High-intensity interval training, Prostate cancer, Maximum oxygen uptake, Fatigue

## Abstract

**Objective:**

This study focused on evaluating whether high-intensity interval training (HIIT) had an effect on aerobic capacity and fatigue among patients with prostate cancer (PCa) and exploring its effect on the immune system of PCa patients.

**Methods:**

To investigate the potential effect of HIIT on patients with prostate cancer, a meta-analysis was carried out. From January 2012 to August 2022, studies that met predefined criteria were searched in the Scopus, PubMed, Web of Science, and EBSCO databases. Analysis of the standardized mean differences was performed using Review Manager 5.4.1 software with a 95% confidence interval.

**Results:**

This review examined a total of 6 articles. There were 215 male patients with PCa involved, and the mean age was 64.4 years. According to the results of the meta-analysis, the HIIT group (*n* = 63) had greater VO_2peak_ (*P*<0.01) than the control group (CON) (*n* = 52) (*P* = 0.30, *I*^2^ = 19% in the heterogeneity test; MD, 1.39 [0.50, 2.27]). Moreover, fatigue was significantly different (*P*<0.01) between the HIIT (*n* = 62) and CON (*n* = 61) groups (*P* = 0.78, *I*^2^ = 0% in the heterogeneity test; SMD, −0.52 [−0.88, −0.16]). Furthermore, among PCa patients, HIIT showed higher efficacy (*P* < 0.01) in decreasing PSA than the CON regimen (*P*=0.22, *I*^2^ = 34% in the heterogeneity test; MD, −1.13 [−1.91, −0.34]).

**Conclusions:**

HIIT improves aerobic capacity, fatigue, and PSA levels among PCa patients but does not significantly affect IL-6 or TNF-α content. Therefore, HIIT may be a novel and potent intervention scheme for PCa patients.

**Supplementary Information:**

The online version contains supplementary material available at 10.1186/s12957-022-02807-8.

## Introduction

Prostatic cancer (PCa) is the second most common male cancer globally [1] and is an important cause of death worldwide [2]. Treatments for PCa vary depending on the disease severity. Radiotherapy (RT) with/without androgen deprivation therapy (ADT) has been extensively adopted in diverse risk groups in line with guidelines from the National Comprehensive Cancer Network [3]. While advancements in RT have decreased cancer mortality, rehabilitative care for PCa remains to be further improved for the increasing number of cancer patients [4]. Cancer patients encounter different, unfavorable, treatment-associated adverse reactions, such as a decrease in aerobic capacity and an increase in fatigue. Cancer-related fatigue (CRF) is generally suggested to be aggravated in 78–89% of cases over the course of RT [5, 6], while exercises involving rehabilitative interventions can mitigate CRF [7].

Recently, high-intensity interval training (HIIT) has attracted much attention because of its short duration and beneficial effects. This regimen involves short bursts of intense activity interspersed by periods of low-intensity exercise or rest. For patients with PCa, the health benefits of HIIT have been widely studied, and HIIT before or after cancer treatment has been demonstrated to markedly enhance aerobic capacity and fatigue in comparison with routine intervention [8, 9]. Typically, continuous HIIT contributes to adapting to cardiorespiratory fitness for adult cancer patients in a short period compared with moderate-intensity training [10]. The above results suggest the critical role of HIIT-induced physiological adaptations in exercise doses ≥80% HR_max_ [11]. Although it has been suggested that HIIT has increasing benefits for adult cancer patients, this remains a controversial opinion. Some studies [12, 13] have indicated that 8 weeks of HIIT training has no effect on aerobic capacity or fatigue in patients with PCa. Therefore, it remains unclear whether HIIT affects aerobic capacity and fatigue in patients with PCa.

This review focused on evaluating whether HIIT had an effect on the aerobic capacity and fatigue of patients with PCa and exploring its effects on the immune system among PCa patients. Our results can shed more light on the application of HIIT in treating PCa.

## Methods

### Protocols and registration

On August 13, 2022, our study protocols were registered at the International Prospective Register of Systematic Reviews (registration number: CRD42022351079). The present review was carried out in line with the PRISMA guidelines (Additional file 1).

### Data sources and study selection

English biomedical databases, including Web of Science, SCOPUS, PubMed, and EBSCO, were searched between January 2012 and August 2022. Keywords for the search were utilized separately or in combination and included the following: “high-intensity intermittent,” “high-intensity interval training,” “prostate cancer,” “training,” and “exercise.” In addition, this study also manually checked reference lists in related systematic reviews and meta-analyses to identify other related articles. Additional file 2 displays more details regarding the study search procedure.

Studies were searched in the above databases by 2 reviewers independently by reading titles and abstracts. Later, data were collected from all studies, including first author, age, publication year, prostate-specific antigen (PSA) level, intervention duration, intervention program, equipment, and major indicators obtained at baseline and endpoint (Table 1). Corresponding authors were contacted to request any missing data via email. Additionally, any disagreement was settled by negotiation with a third reviewer until a consensus was reached.

**Table 1:** Basic information included in the studies

Study	Age (y)	PSA (μg/L)	Duration	HIIT program	CON program	Equipment	Index
Baguley 2022 [9]	65.9±7.8	HIIT: 1.3±1.2 CON: 1.0±1.3	8 weeks; 3x/week	4 sets×(4min 95%HR_peak_: 3min 70%HR_peak_)	Usual care	ergometer	VO_2peak_; fatigue
Djurhuus 2022 [14]	HIIT: 62.5±2.9 CON: 66.8±2.6	HIIT: 11.8±11.8 CON: 15.7±13.9	8 weeks; 4x/week	6 sets×(1min 120%W_peak_: 3min 30%W_peak_)	Usual care	ergometer	VO_2peak_; IL-6; TNF-α; PSA
Kang 2021 [15]	HIIT: 63.9±7.5 CON: 62.8±6.9	HIIT: 6.0±2.3 CON: 8.6±3.5	12 weeks; 3x/week	8 sets×(2min 95%VO_2peak_: 5min 40%VO_2peak_)	Usual care	treadmill	VO_2peak_; PSA
Kang 2022 [16]	HIIT: 63.9±7.5 CON: 62.8±6.9	HIIT: 6.0±2.3 CON: 8.6±3.5	12 weeks; 3x/week	8 sets×(2min 95%VO_2peak_: 5min 40%VO_2peak_)	Usual care	treadmill	Fatigue
Papadopoulos 2021 [13]	HIIT: 62.0±10.4 CON: 62.1±3.2	HIIT: 4.2±2.3 CON: 5.3±2.3	8 weeks; 2x/week	10 sets×(1min 85%HR_peak_: 1min 15W)	Usual care	ergometer	VO_2peak_; IL-6; TNF-α
Piraux 2020 [12]	HIIT: 67.4±8.9 CON: 67.9±7.1	NR	8 weeks; 3x/week	15 sets×(1min 85%HR_peak_: 1min 60%HR_peak_)	Usual care	ergometer	Fatigue

*PSA* prostate-specific antigen, *NR* not reported, *HR*_*peak*_ peak heart rate, *W*_*peak*_ peak power, *VO*_*2peak*_ peak oxygen uptake, *IL-6* interleukin-6, *TNF-α* tumor necrosis factor-α, *HIIT* high-intensity interval training

### Inclusion and exclusion criteria

This review adopted the following criteria to select relevant articles, including randomized controlled trials (RCTs): studies involving PCa patients aged ≥18 years; those regarding HIIT versus routine care; those reporting outcome measures such as peak oxygen uptake (VO_2peak_), fatigue (for any measure used), PAS, tumor necrosis factor-α (TNF-α), and interleukin-6 (IL-6); and those published in English. In addition, case reports, reviews, animal trials, studies without available full texts, or those with insufficient outcome data were excluded. Specifically, articles were screened via 2 steps, namely, title/abstract screening based on our preset inclusion criteria and careful reading of full texts for possibly related articles.

### Assessment of quality

**Fig. 1:**
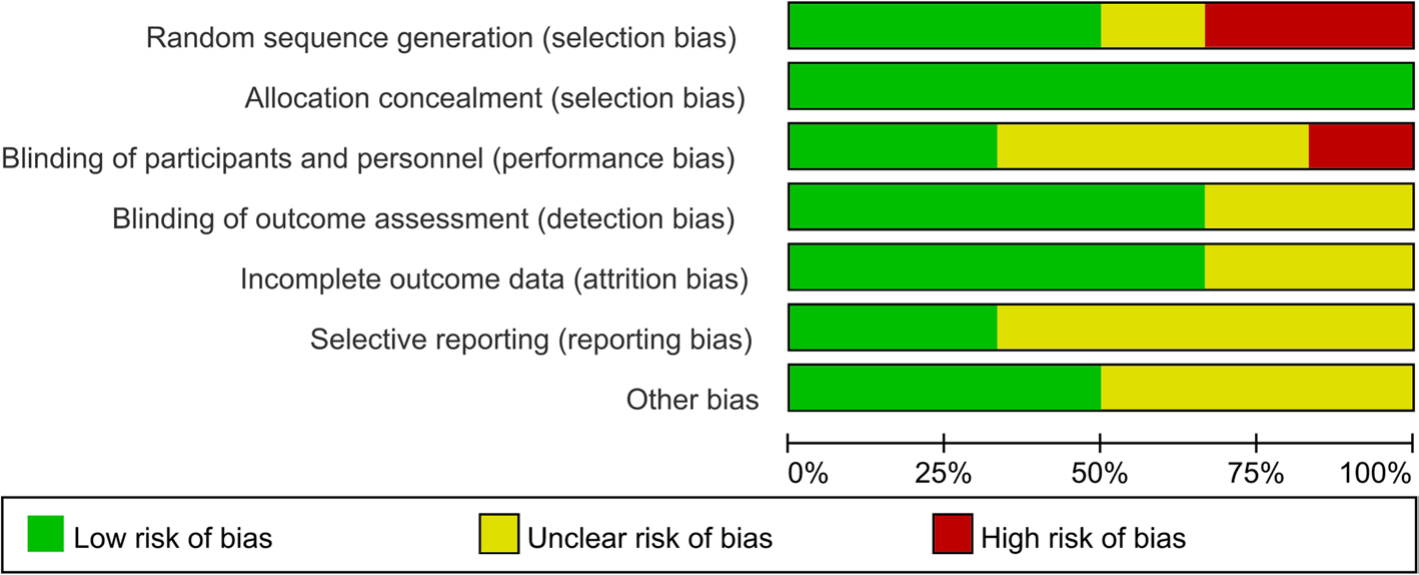
Analysis of risk of bias according to Cochrane Collaboration guideline

This study adopted the Cochrane risk bias assessment approach for evaluating the methodological quality of all enrolled articles. It evaluated the generation of random sequences, concealment of allocation, participant/personnel blinding, outcome measure blinding, selective reporting, insufficient outcome data, and additional biases involved in those articles. Each item was rated as “yes,” “no,” or “unclear.” Figure 1 presents detailed information on the risk of bias analysis.

### Assessment of risk of bias

Each of the included studies was excluded one at a time for sensitivity analysis to analyze the stability of our meta-analysis results. A funnel plot and Egger’s test were adopted to analyze publication bias among the enrolled articles.

### Statistical analysis

Related outcome variables were imported into Review Manager (Version 5.4.1, The Cochrane Collaboration, 2020) for meta-analysis. Continuous outcome variables were examined for all the enrolled articles. The mean difference (MD) was chosen as the effect scale index for identical test methods and units, while the standardized mean difference (SMD) was selected otherwise. Moreover, this study adopted the *I*^2^ statistic for analyzing heterogeneity among diverse articles, where *I*^2^<50% represented the absence of heterogeneity, in which case a fixed-effects model was applied. Finally, a funnel plot was drawn to check the possible bias among articles, and a forest plot was adopted to determine MD and SMD.

## Results

### Article eligibility

**Fig. 2:**
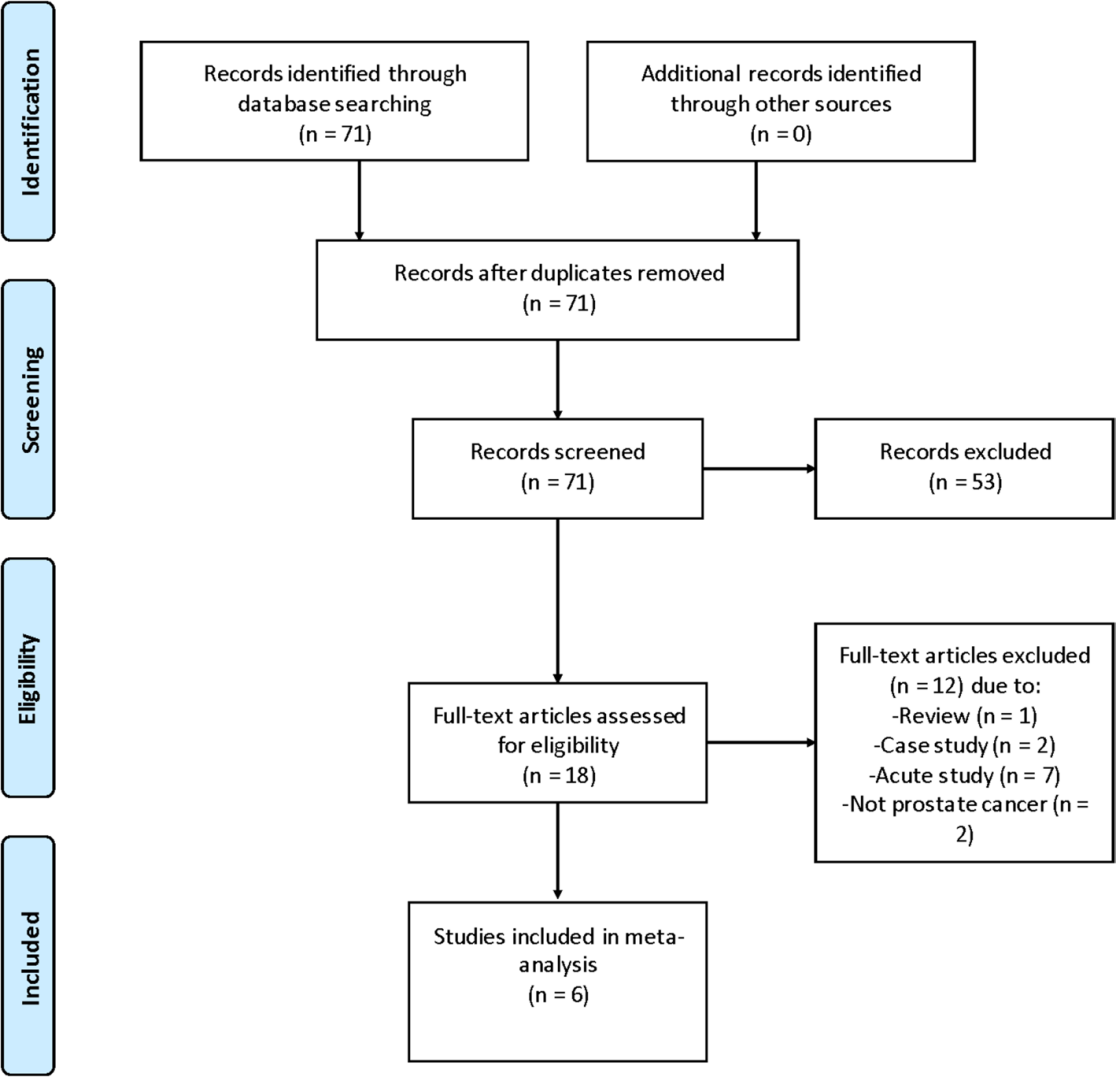
Flow diagram of search results using Preferred Reporting Items for Systematic Reviews and Meta-Analysis

Regarding the search results of the 2 reviewers, Cohen’s kappa coefficient was 0.880. This review examined a total of 6 articles. All studies were RCTs (Fig. 2), all of which satisfied our preset eligibility criteria and mentioned baseline as well as eventual postintervention data. The selected studies were approved by the corresponding institutions. Of them, 4 and 3 evaluated VO_2peak_ and fatigue, respectively, while two evaluated IL-6, TNF-α, and PSA (Table 1). There were 215 male patients involved, and the mean age was 64.4 years. The HIIT intervention duration ranged from 8 to 12 weeks. The intervention program of the CON group was the same as that of the HIIT group. One [16] study used the Functional Assessment of Chronic Illness Therapy (FACIT) to assess fatigue level, and two [9, 12] adopted the quality of life (QOL). Exercise-related side effects were not reported.

### Sensitivity analysis

In this study, separate article exclusion, analysis model alteration, and effect size selection were utilized for sensitivity analysis. Due to the small number of included studies, only VO_2peak_ and fatigue indicators were subject to sensitivity analysis. As a result, the meta-analysis results were not evidently altered following sensitivity analysis, indicating that the results were reliable.

### Quantitative synthesis

**Fig. 3:**
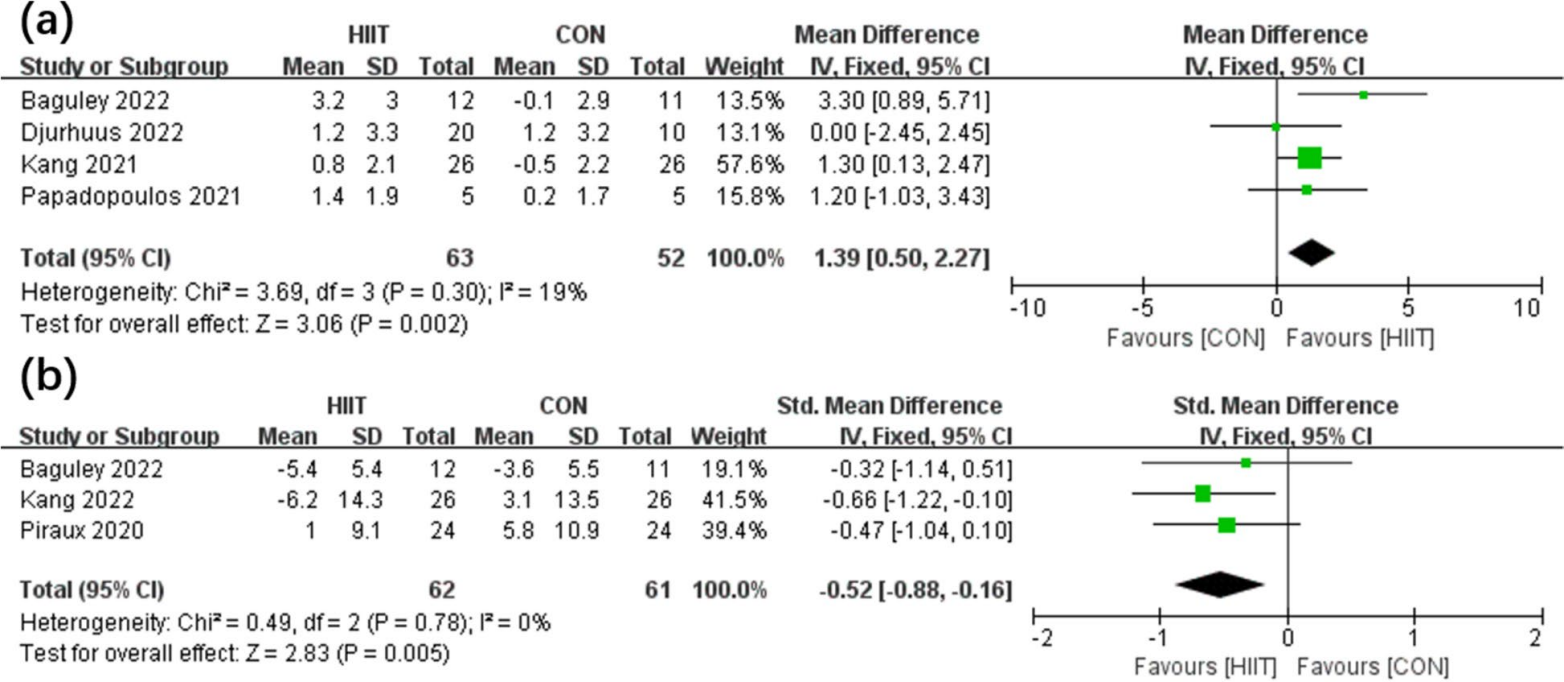
Forest plot illustrating the effects of HIIT vs CON on VO_2peak_ (**a**) and fatigue (**b**)

There were 4 [9, 13–15] and 3 [9, 12, 16] studies comparing the efficacy of the HIIT group and CON group in terms of VO_2peak_ (Fig. 3a) and fatigue, respectively (Fig. 3b). According to the meta-analysis results, the HIIT group (*n* = 63) had enhanced VO_2peak_ (*P*<0.01) compared with the CON group (*n* = 52) (*P* = 0.30, *I*^2^ = 19% according to the heterogeneity test; MD, 1.39 [0.50, 2.27]). Moreover, fatigue was significantly different (*P*<0.01) between the HIIT (*n* = 62) and CON (*n* = 61) groups (*P* = 0.78, *I*^2^ = 0% according to the heterogeneity test; SMD, −0.52 [−0.88, −0.16]).

**Fig. 4:**
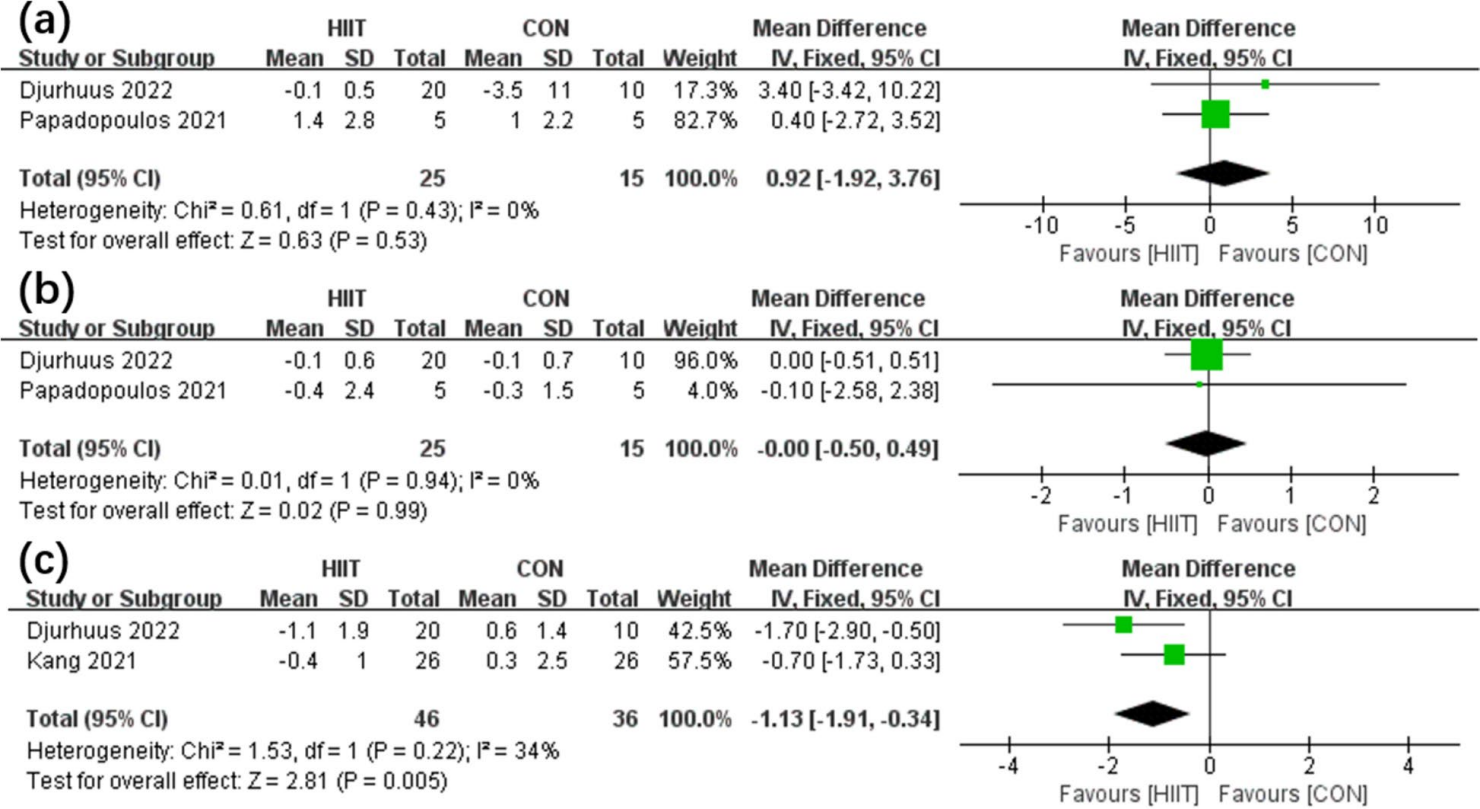
Forest plot illustrating the effects of HIIT vs CON on IL-6 (**a**), TNF-α (**b**), and PSA (**c**)

In addition, there were 2 studies [13, 14] comparing the efficacy of the HIIT group (*n* = 25) and CON group (*n* = 15) in terms of IL-6 (Fig. 4a) and TNF-α (Fig. 4b). The results showed no significant difference in IL-6 (*P*=0.53) or TNF-α (*P*=0.99) (for IL-6: *P* = 0.94, *I*^2^ = 0% according to the heterogeneity test; MD, 0.92 [−1.92, 3.76]; for TNF-α: *P* = 0.43, *I*^2^ = 0% according to the heterogeneity test; MD, −0.00 [−0.50, 0.49]). In addition, there were two studies [14, 15] comparing the efficacy of HIIT (*n* = 46) and CON (*n* = 36) on PSA (Fig. 4c). HIIT showed higher efficacy (*P* < 0.01) in decreasing PSA among PCa patients than CON (*P*=0.22, *I*^2^ = 34% according to the heterogeneity test; MD, −1.13 [−1.91, −0.34]).

### Publication bias

**Fig. 5:**
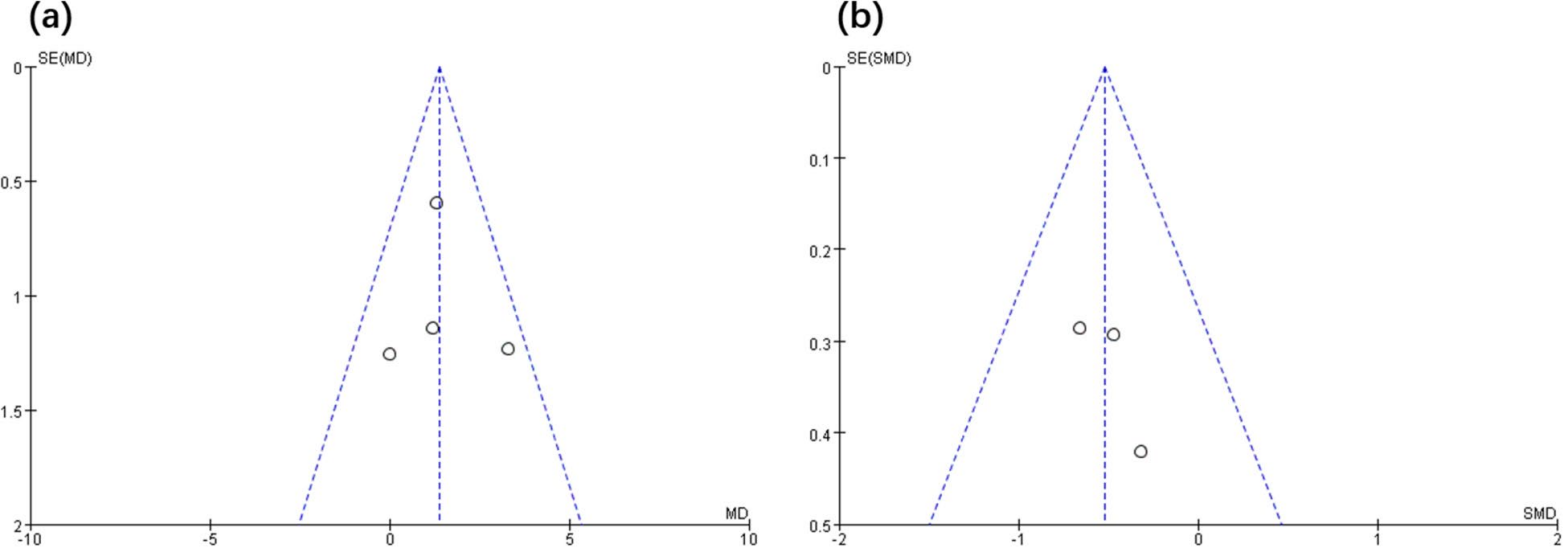
Funnel plot of publication bias for VO_2peak_ (**a**) and fatigue (**b**)

**Fig. 6:**
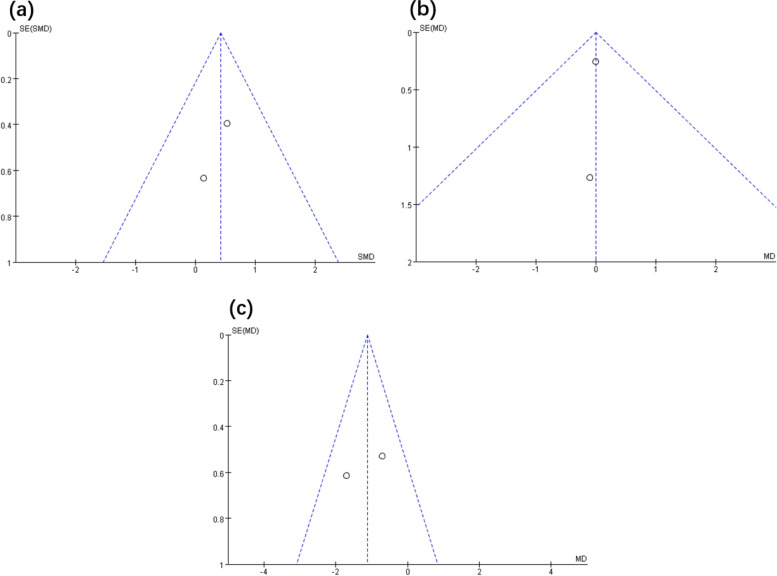
Funnel plot of publication bias for IL-6 (**a**), TNF-α (**b**), and PSA (**c**)

A funnel plot was drawn to analyze publication bias among the enrolled articles. As there were few articles regarding HIIT among PCa patients, only 6 articles were enrolled in this meta-analysis. By adopting the funnel plot, the overall sample size among the enrolled articles approached the minimal requirement, which could partially indicate publication bias. Lu and colleagues [17] suggested that funnel plot analysis was feasible by the use of the small sample size. A funnel plot showing the efficacy of HIIT in terms of VO_2peak_ and fatigue among PCa patients is displayed in Fig. 5. In addition, no evident publication bias was revealed by Egger’s test (VO_2peak_: *P*=0.83, *t*=0.24; fatigue: *P*=0.42, *t*=1.29). Figure 6 presents the funnel plot showing the efficacy of HIIT in terms of IL-6, TNF-α, and PSA among PCa patients.

## Discussion

Although HIIT is often used in the rehabilitation treatment of cancer, few studies have applied it to PCa patients. This review mainly discusses the effect of HIIT on aerobic capacity and fatigue in PCa cases. The secondary endpoint was the effect of HIIT on immune factors among PCa patients. According to this meta-analysis, HIIT significantly improved VO_2peak_, fatigue, and PSA levels over the control treatment, but it did not significantly affect TNF-α or IL-6 content. This result reminded that HIIT might be a novel and potent intervention scheme for PCa patients.

HIIT is defined as either long, repeated (45 s–4 min) bouts of rather high- but not maximal-intensity exercise or short (< 30 s) all-out sprints interspersed with periods of recovery. These varying length efforts combine to create training sessions that last a total of 5–60 min (including recovery intervals) [18]. The four distinct HIIT formats these generate are thought to be important components for inclusion in the periodization of training programs for the development of middle- to long-term physiological adaptation [8]. The exercise intensity in this study was not maximal, and the exercise time was more than 1 min. This indicates that the exercise modes in this study were the traditional HIIT mode but not sprint interval training.

Aerobic capacity is an important physiological index for PCa patients. Specifically, an increase in cardiorespiratory fitness by 3.5 mL/kg/min will reduce cancer-specific mortality by 10% and cardiovascular-related mortality by 25% [19]. Therefore, the elevated VO_2peak_ seen in the present review might provide great benefits for cardiovascular health among PCa patients. The findings of this study are in agreement with those of a prior meta-analysis indicating the safety, feasibility, and efficacy of HIIT in enhancing VO_2peak_ among treated adult cancer patients [10]. For male PCa patients, aerobic exercises can remarkably improve VO_2peak_ [20], and in recent years, 12-week HIIT (8×2 min 85–95%VO_2peak_ treadmill speed and grade, with 2-min active recovery) in the process of active surveillance can dramatically enhance VO_2peak_ in comparison with routine care [15]. These studies support the conclusion of this review. The improvement in VO_2peak_ by HIIT may be related to the adaptation to high physiological load. It has been suggested that HIIT activates complicated molecular interactions within the skeletal muscle to increase oxidative enzyme activities, mitochondrial biogenesis, and angiogenesis [21, 22]. As reported by Laursen and colleagues [23], activation of the AMPK-PGC1α pathway or CAMK-PGC1α has a predominant role in determining cell stimuli to aerobic adaptations, and HIIT more significantly activates AMPK-PGC1α than CAMK-PGC1α. In addition, HIIT stimulates glycogen synthesis [24]. It is possible that the peak lactate level and exhaustion time adaptively increase due to the changes in lactate generation and overload. Consequently, HIIT can effectively improve cardiorespiratory fitness among treated male patients, but such results should be investigated in large and high-quality studies.

It has been reported that exercise can more effectively compensate for fatigue in the treatment course than pharmacological intervention [25]. As confirmed in this review, HIIT better prevented fatigue deterioration than PCa patients receiving usual care. These results conformed to those of prior studies on resistance, aerobic exercise, or their combination among male PCa patients who received radiotherapy intervention [26, 27]. Their radiotherapy regimen was prostate irradiation, received as 68 to 76 Gy in 34 to 38 fractions. Likewise, additional short- (12-week) or long-term (1 year) aerobic training interventions reduce or prevent the worsening of fatigue in patients with PCa [28, 29]. A potential mechanism of exercise interventions in counteracting fatigue is improved exercise capacity [30]. According to our results, the HIIT group had remarkably improved VO_2peak_ in comparison with the CON group. Typically, HIIT is suggested to show higher efficacy in increasing cardiorespiratory fitness than MICT for patients with cancers or cardiometabolic disorders [31, 32]. Consequently, HIIT might promote functional exercise capacity since it enhances oxygen consumption.

Recently, cytokine genetic polymorphisms were found to be related to increased inflammation, cytokine production, and possibly prostate cancer risk [33, 34]. Although these results showed that the inflammation level was not significantly different between the groups, HIIT might suppress the biochemical progression of PCa, consistent with previous results. Currently, a prostate-specific antigen is the best first-step serum marker as a screening test for PCa. It is still the most frequently used oncological marker. Numerous studies have shown that the risk of current and future prostate cancer is directly related to serum PSA [35–37]. Increasing PSA levels are a predictor of a greater risk of adverse pathologic features and worse disease-specific survival [38]. In addition, evidence from a randomized trial further confirmed that PSA testing reduces both metastatic disease and prostate cancer-specific mortality [39]. As reported by Kang and colleagues [15], HIIT exercise at 95% VO_2peak_ was used for a 12-week period, thrice a week. According to their results, HIIT promoted cardiorespiratory fitness while reducing PSA velocity, PSA content, and PCa cell proliferation among male localized PCa patients receiving active surveillance. As indicated by one exploratory exercise article carried out among PCa patients receiving active surveillance, PSA content was not changed after long-term, home-based moderate-intensity exercise intervention [40]. The reason for this difference may lie in the difference in exercise intensity. In contrast, our adopted exercise program placed greater emphasis on short-term (8–12 weeks), high-intensity exercise (namely, 85–95% HR_max_), which induced more physiological alterations (such as cytotoxic immunocyte mobilization and sympathetic activation) [41, 42]. Based on the above results, HIIT might be necessary for producing changes in the biochemical outcomes of PCa. The biological mechanisms of the effects of exercise on prostate cancer remain unclear. One plausible mechanism is the enhanced immunosurveillance after exercise training or even during a single bout of exercise [43, 44]. Specifically, exercise can mobilize cytotoxic natural killer cells into circulating blood and can redistribute these cells to tumor cells with assistance from exercise-induced increases in circulating norepinephrine and IL-6 [41]; this process appears to require endurance exercise at high intensity [45]. Other possible explanations include exercise-based suppression of prostate cancer progression via modulation of systemic inflammatory mediators [46], metabolic biomarkers [47], and tumor vascularization and perfusion [48]. More research in active surveillance clinical settings is necessary to identify the biophysiological associations between exercise and prostate cancer [49] and to further explore potential tumor-related biomarkers [50].

In addition, the progression of patients with PCa can be divided into early and advanced stages. Although the effect of HIIT application in different periods is unclear, according to the current research, the early use of exercise intervention may not affect the progression of prostate cancer [51]. However, the use of exercise intervention in any period can yield certain benefits, especially in the advanced stage, and the use of exercise intervention can significantly improve the quality of life, walking ability, and mortality of patients [52, 53].

However, there were still many limitations in this study. First, this study only targeted PCa patients but did not include other cancer patients. Second, since HIIT has only been applied to PCa patients in recent years (2020–2022), few studies were included. Therefore, our findings must be interpreted cautiously and should be supplemented by more studies in the future. In addition, no other forms of exercise were compared with HIIT, such as moderate-intensity continuous training or resistance training. Therefore, it was impossible to determine which form of exercise was more effective as an intervention for PCa patients. Finally, the observed indicators were not comprehensive, and changes in other inflammatory factors and anti-inflammatory factors could not be observed due to the low number of included studies. Future studies should be conducted to analyze how HIIT affects the quality of life or other physiological indicators of PCa. However, we still suggest that doctors use HIIT as a means to intervene in the non-drug treatment of prostate cancer patients after determining the exercise risk of the patient.

## Conclusion

HIIT improves aerobic capacity, fatigue, and PSA levels among PCa patients but does not significantly affect IL-6 or TNF-α content. Therefore, HIIT may be a novel and potent intervention scheme for PCa patients.

## Supplementary Information

**Additional file 1.** PRISMA 2009 checklist.

**Additional file 2.** Search strategy.

## Data Availability

All data are available from the corresponding authors.
