# Supplementary material for: Effect of high-intensity interval training on aerobic capacity and fatigue among patients with prostate cancer: a meta-analysis
Source: World J Surg Oncol. 2022 Oct 19;20:348. doi: 10.1186/s12957-022-02807-8 (PMC9580114; doi:10.1186/s12957-022-02807-8)
Supplement: Supplementary file 2 — Additional file 2. Search strategy. [file 12957_2022_2807_MOESM2_ESM.docx]

**Appendix A**

Search strategy

| Databases | Search strategy | Result  (Approximately) |
| --- | --- | --- |
| Scopus | #1: Title-Abs-Key (“High intensity interval” or “High intensity intermittent”)  #2: Title-Abs-Key (Prostate cancer)  #3: Title-Abs-Key (Exercise or Training)  #4: #1 and #2 and #3  Limiters - Published Date: 20120101-20220807 | 5,019  129,682  1,098,931  23 |
| Pubmed | #1: [Title/Abstract] “High intensity interval” or “High intensity intermittent”  #2: [Title/Abstract] Prostate cancer  #3: [Title/Abstract] Exercise or Training  #4: #1 and #2 and #3  Filters: Publication date from 2012/01/01 to 2022/8/07 | 3,373  78,409  415,095  13 |
| Web of Science | #1: TOPIC: (“High intensity interval” or “High intensity intermittent”)  #2: TOPIC: (Prostate cancer)  #3: TOPIC: (Exercise or Training)  #4: #1 and #2 and #3  Refined by: PUBLICATION YEARS: (20220807-20120101)  Indexes=SCI-EXPANDED, SSCI, CCR-EXPANDED, | 5,286  170,341  1,339,847  18 |
| EBSCO | #1: Abstract: (“High intensity interval” or “High intensity intermittent”)  #2: Abstract: (Prostate cancer)  #3: Abstract: (Exercise or Training)  #4: #1 and #2 and #3  Year: 20010101-20220807 | 4,897  13,587  987,568  17 |
